# Supplementary material for: A Critical View of the Application of the APEX Software (Aqueous Photochemistry of Environmentally-Occurring Xenobiotics) to Predict Photoreaction Kinetics in Surface Freshwaters
Source: Molecules. 2019 Dec 18;25(1):9. doi: 10.3390/molecules25010009 (PMC7017383; doi:10.3390/molecules25010009)
Supplement: Supplementary file 1 [file molecules-25-00009-s001.zip › 3.2.4_gcc-4.4.0.zip!/3.2.4_gcc-4.4.0/3.2.4_gcc-4.4.0/doc/octave/html/interpreter/Commands-For-History.html]

Commands For History - Untitled


Next: Customizing readline,
Previous: Commands For Completion,
Up: Command Line Editing


---

#### 2.4.5 Commands For Manipulating The History

Octave normally keeps track of the commands you type so that you can
recall previous commands to edit or execute them again. When you exit
Octave, the most recent commands you have typed, up to the number
specified by the variable `history_size`, are saved in a file.
When Octave starts, it loads an initial list of commands from the file
named by the variable `history_file`.

Here are the commands for simple browsing and searching the history
list.

`<LFD>` `<RET>`: Accept the current line regardless of where the cursor is. If the line is non-empty, add it to the history list. If the line was a history line, then restore the history line to its original state. `C-p`: Move `up' through the history list. `C-n`: Move `down' through the history list. `M-<`: Move to the first line in the history. `M->`: Move to the end of the input history, i.e., the line you are entering! `C-r`: Search backward starting at the current line and moving `up' through the history as necessary. This is an incremental search. `C-s`: Search forward starting at the current line and moving `down' through the history as necessary.

On most terminals, you can also use the up and down arrow keys in place
of `C-p` and `C-n` to move through the history list.

In addition to the keyboard commands for moving through the history
list, Octave provides three functions for viewing, editing, and
re-running chunks of commands from the history list.

— Command: **history** options  
> If invoked with no arguments, `history` displays a list of commands
> that you have executed. Valid options are:
>
> `-w` file: Write the current history to the file file. If the name is omitted, use the default history file (normally ~/.octave\_hist). `-r` file: Read the file file, replacing the current history list with its contents. If the name is omitted, use the default history file (normally ~/.octave\_hist). n: Display only the most recent n lines of history. `-q`: Don't number the displayed lines of history. This is useful for cutting and pasting commands using the X Window System.
>
> For example, to display the five most recent commands that you have
> typed without displaying line numbers, use the command
> `history -q 5`.

— Command: **edit\_history** [first] [last]  
> If invoked with no arguments, `edit_history` allows you to edit the
> history list using the editor named by the variable `EDITOR`. The
> commands to be edited are first copied to a temporary file. When you
> exit the editor, Octave executes the commands that remain in the file.
> It is often more convenient to use `edit_history` to define functions
> rather than attempting to enter them directly on the command line.
> By default, the block of commands is executed as soon as you exit the
> editor. To avoid executing any commands, simply delete all the lines
> from the buffer before exiting the editor.
>
> The `edit_history` command takes two optional arguments specifying
> the history numbers of first and last commands to edit. For example,
> the command
>
> ```
>           edit_history 13
> ```
>
> extracts all the commands from the 13th through the last in the history
> list. The command
>
> ```
>           edit_history 13 169
> ```
>
> only extracts commands 13 through 169. Specifying a larger number for
> the first command than the last command reverses the list of commands
> before placing them in the buffer to be edited. If both arguments are
> omitted, the previous command in the history list is used.
>
>
>
> **See also:** run\_history.

— Command: **run\_history** [first] [last]  
> Similar to `edit_history`, except that the editor is not invoked,
> and the commands are simply executed as they appear in the history list.
>
>
>
> **See also:** edit\_history.

Octave also allows you customize the details of when, where, and how history
is saved.

— Built-in Function: val = **saving\_history** ()  
— Built-in Function: old\_val = **saving\_history** (new\_val)  
> Query or set the internal variable that controls whether commands entered
> on the command line are saved in the history file.
>
>
>
> **See also:** history\_file, history\_size, history\_timestamp\_format\_string.

— Built-in Function: val = **history\_file** ()  
— Built-in Function: old\_val = **history\_file** (new\_val)  
> Query or set the internal variable that specifies the name of the
> file used to store command history. The default value is
> ~/.octave\_hist, but may be overridden by the environment
> variable `OCTAVE_HISTFILE`.
>
>
>
> **See also:** history\_size, saving\_history, history\_timestamp\_format\_string.

— Built-in Function: val = **history\_size** ()  
— Built-in Function: old\_val = **history\_size** (new\_val)  
> Query or set the internal variable that specifies how many entries
> to store in the history file. The default value is `1024`,
> but may be overridden by the environment variable `OCTAVE_HISTSIZE`.
>
>
>
> **See also:** history\_file, history\_timestamp\_format\_string, saving\_history.

— Built-in Function: val = **history\_timestamp\_format\_string** ()  
— Built-in Function: old\_val = **history\_timestamp\_format\_string** (new\_val)  
> Query or set the internal variable that specifies the format string
> for the comment line that is written to the history file when Octave
> exits. The format string is passed to `strftime`. The default
> value is
>
> ```
>           "# Octave VERSION, %a %b %d %H:%M:%S %Y %Z <USER@HOST>"
> ```
>
>
>
>
> **See also:** strftime, history\_file, history\_size, saving\_history.

— Built-in Function: val = **EDITOR** ()  
— Built-in Function: old\_val = **EDITOR** (new\_val)  
> Query or set the internal variable that specifies the editor to
> use with the `edit_history` command. The default value is taken from
> the environment variable `EDITOR` when Octave starts. If the
> environment variable is not initialized, `EDITOR` will be set to
> `"emacs"`.
>
>
>
> **See also:** edit\_history.
